# A novel inactivated whole-cell *Pseudomonas aeruginosa* vaccine that acts through the cGAS-STING pathway

**DOI:** 10.1038/s41392-021-00752-8

**Published:** 2021-10-01

**Authors:** Cuicui Ma, Xiao Ma, Boguang Jiang, Hailong Pan, Xueyuan Liao, Li Zhang, Wenfang Li, Yingjie Luo, Zhixue Shen, Xingjun Cheng, Mao Lian, Zhenling Wang

**Affiliations:** 1 State Key Laboratory of Biotherapy and Cancer Center, West China Hospital, Sichuan University, and Collaborative Innovation Center of Biotherapy, Chengdu, 610041 China; 2 National Institutes for Food and Drug Control (NIFDC), Beijing, 100050 China; 3 Department of Quality Management, China National Biotec Group Company Limited, Beijing, 100020 China

**Keywords:** Vaccines, Infection

## Abstract

*Pseudomonas aeruginosa* infection continues to be a major threat to global public health, and new safe and efficacious vaccines are needed for prevention of infections caused by *P. aeruginosa*. X-ray irradiation has been used to prepare whole-cell inactivated vaccines against *P. aeruginosa* infection. However, the immunological mechanisms of X-ray-inactivated vaccines are still unclear and require further investigation. Our previous study found that an X-ray-inactivated whole-cell vaccine could provide protection against *P. aeruginosa* by boosting T cells. The aim of the present study was to further explore the immunological mechanisms of the vaccine. Herein, *P. aeruginosa* PAO1, a widely used laboratory strain, was utilized to prepare the vaccine, and we found nucleic acids and 8-hydroxyguanosine in the supernatant of X-ray-inactivated PAO1 (XPa). By detecting CD86, CD80, and MHCII expression, we found that XPa fostered dentritic cell (DC) maturation by detecting. XPa stimulated the cGAS-STING pathway as well as Toll-like receptors in DCs in vitro, and DC finally underwent apoptosis and pyroptosis after XPa stimulation. In addition, DC stimulated by XPa induced CD8^+^ T-cell proliferation in vitro and generated immunologic memory in vivo. Moreover, XPa vaccination induced both Th1 and Th2 cytokine responses in mice and reduced the level of inflammatory factors during infection. XPa protected mice in pneumonia models from infection with PAO1 or multidrug-resistant clinical isolate W9. Chronic obstructive pulmonary disease (COPD) mice immunized with XPa could resist PAO1 infection. Therefore, a new mechanism of an X-ray-inactivated whole-cell vaccine against *P. aeruginosa* infection was discovered in this study.

## Introduction

*Pseudomonas aeruginosa* (*P. aeruginosa*) infection is a major threat to global public health. As a Gram-negative bacteria and prevalent opportunistic pathogen, *P. aeruginosa* can cause severe nosocomial infections in various body sites, including the lower respiratory tract, surgical and burn wounds, urinary tract, and cornea.^1–3^ The risks of *P. aeruginosa* infection are increased in immunocompromised hosts, such as chronic obstructive pulmonary disease (COPD) patients,^4^ mechanically ventilated patients,^5^ and those with cystic fibrosis.^6^ In the 2019 Antibiotic Resistance Threats Report from the Centers for Disease Control (CDC), *P. aeruginosa* was listed as a critical pathogen for which new antimicrobial agents are urgently needed.^7^ *P. aeruginosa* has always been considered a difficult target for antimicrobial chemotherapy due to its comprehensive mechanism of resistance, including target mutations, active efflux, the expression of antibiotic-modifying enzymes, and biofilm formation.^8^ With the capacity to form concomitant biofilms,^9^ which (i) shield bacteria from host immunity^10^ and (ii) subvert the innate immune system through host cells,^11^ the treatment of *P. aeruginosa infection* faces greater challenges.

Due to its comprehensive mechanisms that confer *P. aeruginosa* with drug resistance, vaccines capable of preventing *P. aeruginosa* infection should not alter the immunological characteristics of the organism against which an effective, vaccine-induced immune response is targeted. In the past several decades, enormous efforts^12^ have been focused on the vaccine development, but no approved vaccine is available for application to treat *P. aeruginosa* infection.^2^ Subunit vaccines seem to fail to induce an even stronger immune response than attenuated live vaccines.^13^ The protective efficacy of vaccines based on flagellin was limited in clinical isolates from CF patients.^14^ The serological diversity of the pili^15^ blocks the development of vaccines based on pili.^16^ Live vaccines typically provide better immunity than killed or subunit vaccines owing to their mimicry of a natural infection, but they are generally associated with safety concerns.^17^ Our previous study reported that X-ray-inactivated *P. aeruginosa* is a potential vaccine candidate that can induce cellular and humoral immune responses. Recently, we found that X-ray irradiation induced nucleic acid release of *P. aeruginosa*,^18^ but the mechanism by which nucleic acids are involved in the immune response remains undetermined.

The cyclic GMP–AMP synthase (cGAS)-STING pathway^19^ and Toll-like receptor (TLR) family^20–22^ play key roles in innate immune responses by recognizing pathogen-associated molecular patterns, including nucleic acids of bacteria or viruses, lipopolysaccharides (LPS), and flagellin. Studies have shown that the cGAS-STING pathway is vital in the innate immune response to cytosolic DNA by inducing type-I interferon (IFN-I) production against DNA virus/bacterial infection.^23^ Dendritic cells (DCs) are the most potent antigen-presenting cells (APCs) that bridge innate and adaptive immunity in vivo, and their maturation can maintain the balance of tolerance and immunity.^24,25^ STING in DCs induces IFN-I responses to activate immediate innate defenses and direct subsequent effector T-cell responses.^26^ Many studies have found that STING agonizts were effective in cancer treatments.^27,28^ The development of such strategies for vaccines against *P. aeruginosa* will lead to enhanced protection that might directly benefit patients.

Our previous study reported that a whole-cell vaccine inactivated by X-ray irradiation had potential to prevent *P. aeruginosa* infection. The vaccine could protect mice from infection with homotypic and heterotypic *P. aeruginosa* by boosting the cellular immune response, mainly CD4^+^ T cells.^29^ *P. aeruginosa* generally activates host humoral immunity during infection, which cannot boost specific T-cell responses or provoke immunological memory. In this study, considering that T cells cannot be activated without APCs, we aimed to explore how the XPa vaccine impacts DC function and specific T-cell activation through the cGAS-STING pathway and other receptors.

## Results

### X-ray irradiation induced nucleic acid release of PAO1

**Fig. 1:**
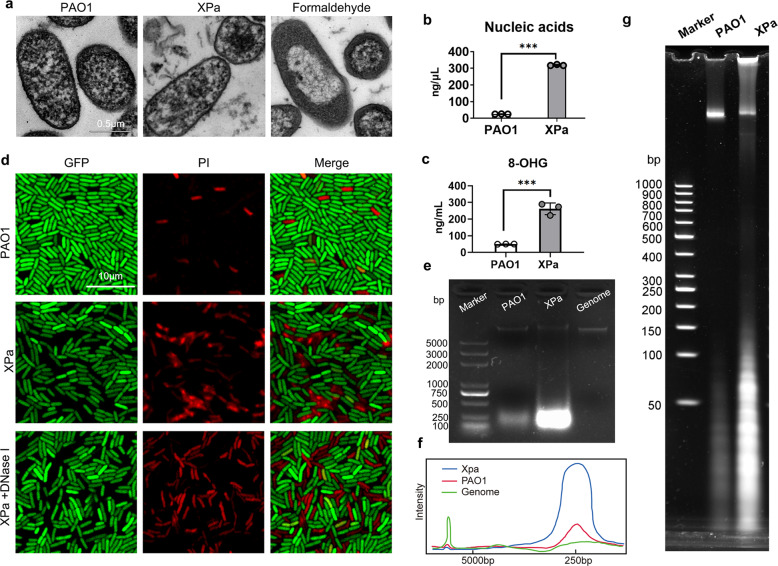
Morphology and biochemical characterization of XPa. **a** Transmission electron microscopy of PAO1, XPa (X-ray-treated PAO1), and formaldehyde-treated PAO1. Determination of nucleic acid (**b**) and 8-OHG (**c**) concentrations in the supernatant. **d** PI-stained PAO1-GFP after X-ray irradiation (XPa) and PAO1, and DNase I-treated XPa. **e** Nucleic acid size distribution in PAO1 and XPa (980 Gy) supernatants and the PAO1 genome were analyzed via agarose gel electrophoresis in the presence of a 2000-bp DNA ladder as a marker. **f** Molecular weight distribution curves according to **e**. **g** The supernatants of the PAO1 and XPa groups were separated on a 4–20% polyacrylamide TBE gel (Invitrogen, EC62255BOX), and run in 1× TBE buffer for 30 min. The gel was stained with SYBR Gold Nucleic Acid Gel Stain and detected using an enhanced chemiluminescence system (iBright CL1000, Thermo Fisher). Data were analyzed using an unpaired *t*-test. ***P < 0.001

We conducted transmission electron microscopy to observe the shape of PAO1 after X-ray irradiation. The PAO1 cells showed a consistent electron density, and DNA molecules and electron-light material in the picture distributed randomly in almost all parts of the cells (Fig. 1a). In the formaldehyde group, there was a large electron-light area in the center of the cell, indicating accumulation of DNA molecules.^30^ Compared with PAO1, XPa showed a similar structure with decreased electron density, which suggests something lost.

Our previous study showed that X-ray irradiation induced nucleic acid release of PAO1.^18^ X-rays induced the production of the reactive form of oxygen, which causes oxidative damage to nucleic acids.^31^ We tested the concentrations of nucleic acids and 8-hydroxyguanosine (8-OHG) in the supernatant of the XPa and PAO1 groups (Fig. 1b, c). The concentration of nucleic acids was significantly increased from 25.4 ± 0.5 ng/μL to 319.4 ± 3.6 ng/μL (approximately 13-fold) after exposure to X-ray irradiation. The concentration of 8-OHG was significantly increased from 50.02 ± 0.63 ng/mL to 261.90 ± 35.18 ng/mL (approximately 5-fold) after irradiation. Next, to locate and visualize the released nucleic acids, we performed propidium iodide (PI) staining of PAO1-expressing GFP (Fig. 1d). The appearance of a red signal outside the cell indicated the presence of nucleic acids. Compared with PAO1, nucleic acids (red signal) in the XPa group were significantly more abundant (Fig. 1d). Furthermore, we treated the XPa sample with DNase I, and found that the nucleic acids surrounding the cell disappeared. Thus, we hypothesized that X-ray irradiation induced the nucleic acid release of PAO1. Disappearance of the GFP signal (green) was noted in the PI-labeled cells, and few of the cells were labeled with PI (red) and GFP (green), showing an overlay (yellow) of signals. This result suggests that the viability of PAO1-GFP might be reduced after X-ray irradiation.

The fragment sizes of nucleic acids were analyzed via gel electrophoresis (Fig. 1e, f), and the genomic DNA (approximately 6.5 Mbp) of PAO1 was used as a control. Both PAO1 and XPa samples contained a small amount of genomic DNA, but the 250-bp band for PAO1 was blurred, while that in the XPa lane showed high intensity. Furthermore, the nucleic acids were resolved on a 4–20% polyacrylamide TBE gel (Fig. 1g). The distribution of the small-fragment nucleic acids was under 100 bp. The results indicated that the nucleic acids in the cells were damaged by X-ray irradiation and released into the extracellular environment (Fig. 1b).

### XPa modulates DC function

**Fig. 2:**
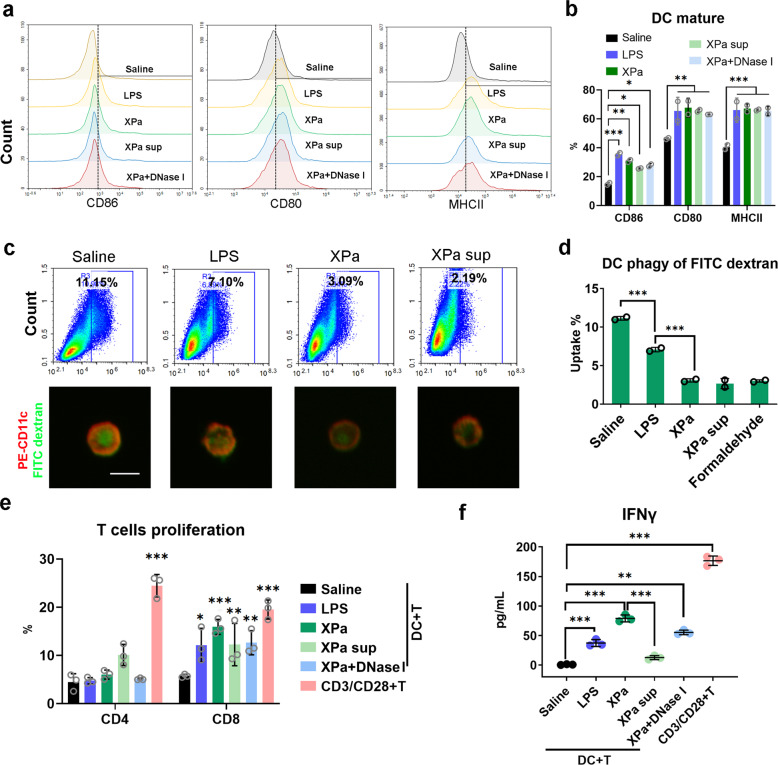
Effect of XPa on DC functions. **a** and **b** DC maturation. DCs were stimulated with saline, XPa (10 MOI) and LPS (100 ng), XPa sup (same volume of XPa), and XPa + DNase I (10 MOI) for 24 h, and then collected for flow cytometry analysis. **b** The percentages of CD86^+^, CD80^+^, and MHCII^+^ among CD11c^+^ cells. **c** Uptake of FITC–dextran by BMDCs subjected to the different treatments. Scale bar=50 μm. **d** Data analysis of **c**. **e** The stimulated DCs (saline; XPa (10 MOI) and LPS (100 ng); XPa sup (same volume as XPa) and XPa + DNase I (10 MOI) for 24 h) were cocultured with the CFSE-stained T cells at a ratio of 1:10 after 72 h, with CD3/CD28 as a positive control. * indicates significant from the saline control. **f** The IFN-γ concentration in the culture medium. Data were analyzed via two-way ANOVA. No letter is present if no significant difference was observed. *P < 0.05, **P < 0.01, ***P < 0.001

We assessed bone marrow-derived dendritic cell (BMDC) functions after XPa stimulation (Fig. 2). DCs significantly upregulated the CD86, CD80, and MHCII expression upon stimulation with Xpa (CD86 30.81 ± 1.21%, CD80 67.68 ± 6.58%, and MHCII 67.18 ± 3.17%), XPa supernatant (XPa sup) (CD86 25.64 ± 0.36%, CD80 65.61 ± 1.06%, and MHCII 66.12 ± 0.66%), XPa + DNase I (CD86 27.81 ± 1.21%, CD80 63.02 ± 0.11%, and MHCII 65.37 ± 3.22%), and the positive control LPS (100 ng/mL) (CD86 35.52 ± 1.40%, CD80 65.35 ± 9.58%, and MHCII 65.95 ± 8.45%) compared with saline (CD86 15.00 ± 1.11%, CD80 46.28 ± 0.75%, and MHCII 40.40 ± 2.19%) (Fig. 2a, b). Mature DCs exhibited downregulated phagocytic capacity, and we found that the antigen-(FITC–dextran) uptake capacity of DCs was reduced after treatment with XPa (3.09 ± 0.21%) and XPa sup (2.19 ± 0.05%) compared with saline (11.15 ± 0.24%) and LPS (100 ng/mL) (7.10 ± 0.29%) (Fig. 2c, d). Normally, DCs present the processed antigens to T cells and elicit T-cell proliferation during an antigen-specific T-cell response.^23^ The proliferation of CD4^+^ and CD8^+^ T cells was analyzed via flow cytometry (Fig. 2e), and IFN-γ production (Fig. 2f) was measured after 72 h of coculture. CD8^+^ T cells showed significant proliferation in the XPa (15.93 ± 1.57%), XPa sup (12.26 ± 4.40%), XPa + DNaseI (12.64 ± 2.50%), and positive-control CD3/CD28 (19.54 ± 1.97%) groups compared with the saline group (5.75 ± 0.34%) (Fig. 2e). The level of IFN-γ in the LPS (37.45 ± 5.85 pg/mL), XPa (79.28 ± 5.35 pg/mL), XPa sup (12.85 ± 3.40 pg/mL), XPa + DNase I (55.42 ± 3.82 pg/mL), and the CD3/CD28 + T-cell (176.67 ± 7.95 pg/mL) groups was significantly higher than that in the saline group (1.08 ± 0.67 pg/mL) (Fig. 2f).

### XPa-activated STING in DCs

**Fig. 3:**
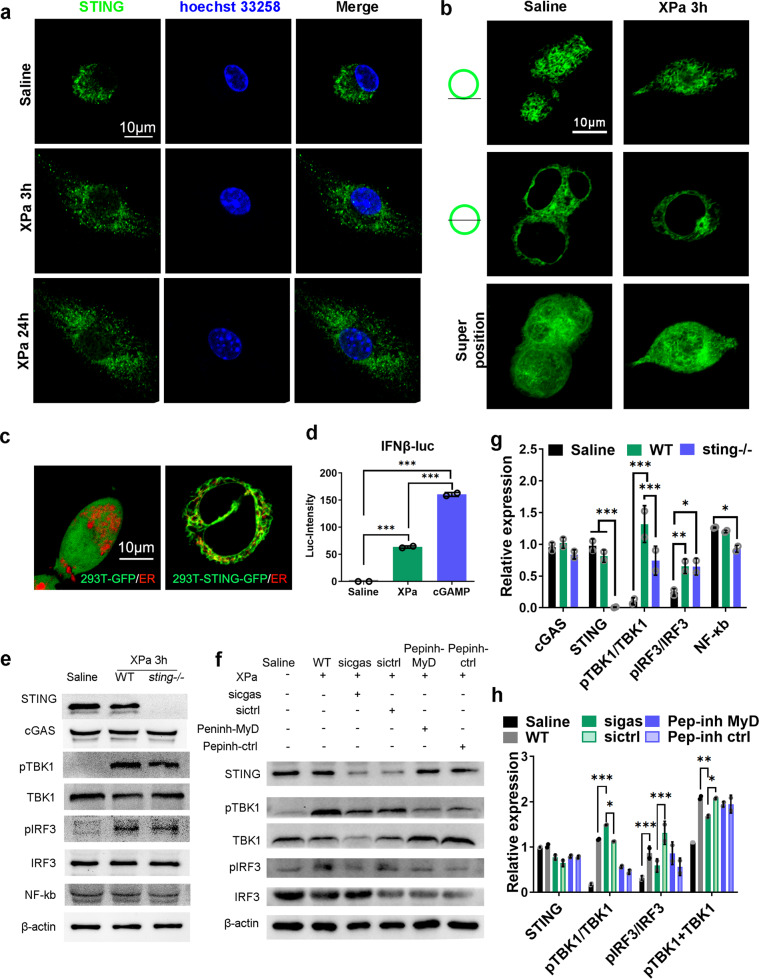
XPa activated STING in BMDCs. **a** STING immunofluorescence staining of BMDCs stimulated with XPa for 3 h. **b** Lentivirus-infected 293 T cells stably expressing STING-GFP were treated with XPa for 3 h. **c** Lentivirus-infected 293 T cells stably expressing STING-GFP or GFP were stained with ER tracker (Invitrogen). The column graph shows the luciferase intensities of 293T-STING-GFP cells (transfected with IFN-β-promotor–luc plasmid) stimulated with XPa and the STING agonist—cGAMP (Sigma, sml1229). **d** Western blot analysis of related proteins in cGAS-STING pathway. **e** Western blot of BMDCs stimulated by XPa for 3 h after *sicgas* or MyD88 inhibitor (Pepinh-MyD) treatment. *sictrl* or Pepinh-ctrl as controls in the experiment. **f** Grayscale analysis of the western blot bands of **d**, *n* = 2. **g** Grayscale analysis of the western blot bands of **e**. Data were analyzed using two-way ANOVA test. *P < 0.05, **P < 0.01, ***P < 0.001. No letter is present if no significant difference was observed

In this study, the movement of STING was observed via immunofluorescence staining in BMDCs stimulated by XPa. DCs incubated with XPa showed a modification in cell morphology (forming pseudopodia) (Fig. 3a, Supplementary movies 1 and 2). To further demonstrate this phenomenon, we constructed 293T-STING-GFP cells via lentivirus infection and stimulated the cells with XPa for 3 h (Fig. 3b). We found condensed green fluorescence around the cell nucleus after 3 h of XPa treatment (data not shown). In 293T-STING-GFP, STING was localized to the endoplasmic reticulum (ER) with GFP as a control (in transverse section) (Fig. 3c, Supplementary movies 3 and 4). To examine the activation of the STING pathway, an IFN-β-promoter–firefly luciferase (Luc) plasmid was transfected into 293T-STING-GFP. The luciferase intensities were detected by the Luciferase Reporter Gene Assay Kit with a fluorescence microplate reader. 293T-STING-GFP-Luc cells generated a high luciferase intensity (160.5 ± 3.54) when stimulated by 16.5 μg/mL cGAMP (STING agonist), and an intensity of 63.5 ± 2.12 when stimulated by XPa, approximately 40% of that generated by cGAMP. This result indicates that XPa activates the cGAS-STING pathway.

### XPa activated the cGAS-STING pathway

cGAS-STING pathway-related proteins were tested via western blot analysis (Fig. 3e, g). The results showed that the pTBK1/TBK1 (1.34 ± 0.05) and pIRF3/IRF3 (0.61 ± 0.04) ratios were increased after stimulation with XPa compared with saline (pTBK1/TBK1 0.10 ± 0.12, pIRF3/IRF3 0.19 ± 0.01). When STING was absent, the ratio of pTBK1/TBK1 (0.73 ± 0.02) was decreased compared with that in the wild type (WT) and after XPa treatment.

**Fig. 4:**
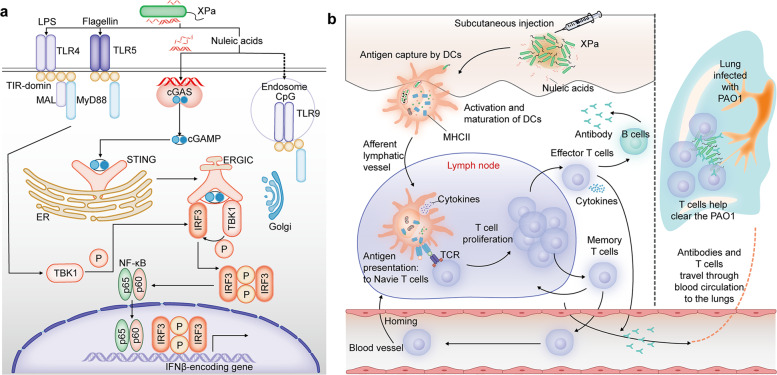
Activated pathways in DCs stimulated by XPa and the immune response during immunization and infection. **a** The activated pathways in DCs induced by XPa. TLR4 recognizes LPS, TLR5 recognizes the flagellin protein, the cGAS-STING pathway is stimulated by nucleic acids, and TLR9 recognizes CpG (which was not detected in XPa). MyD88 can activate IRF3 through the TBK1 protein in TLR4 signaling, thus increasing downstream gene expression (MyD88–TBK1-independent genes). **b** The immune response during immunization and infection. Upon encountering XPa, DCs are rapidly activated and become mature. Mature DCs migrate to the lymph nodes where DCs present antigens to T cells. Following antigen encounter, T cells are activated, proliferate, and differentiate into effector and memory T cells (TCMs and TEMs). When mice are exposed to PAO1 challenge, TEMs help rapidly clear the bacteria

Because the whole-cell vaccine contains multiple antigens, complex pathways are involved in the mechanism of XPa, including the cGAS-STING pathway and TLRs, such as TLR4 and TLR5. MyD88 is a crucial adapter molecule that is downstream of TLRs,^32^ thus, the referred TLRs are MyD88-dependent pathways. Therefore, we divided the immune receptors into MyD88-independent pathways (cGAS-STING) and MyD88-dependent pathways (TLRs) (Fig. 4). In this study, Pepinh-MyD (MyD88-inhibitor peptide) and *sicgas* (siRNA of *cgas*.) were used to analyze the impacts of TLRs and the cGAS-STING pathway in XPa-activated BMDCs. The expression of pTBK1 and pIRF3 was analyzed via Western blotting, as shown in Fig. 3f, h. *sicgas* but not *sictrl* (negative control of *sicgas*) significantly increased the pTBK1/TBK1 ratio (*sicgas* 1.49 ± 0.01, *sictrl* 1.12 ± 0.01) and reduced the pTBK1 + TBK1 ratios (*sicgas* 1.68 ± 0.03, *sictrl* 2.07 ± 0.02), pIRF3/IRF3 (*sicgas* 0.59 ± 0.15, *sictrl* 1.31 ± 0.26) compared with WT (pTBK1/TBK1 1.17 ± 0.02, pTBK1 + TBK1 2.09 ± 0.05, and pIRF3/IRF3 0.86 ± 0.16). However, both Pepinh-MyD and Pepinh-ctrl (negative control of Pepinh-MyD) reduced the pTBK1/TBK1 ratios (Pepinh-MyD 0.56 ± 0.03, Pepinh-ctrl 0.45 ± 0.05) and pIRF3/IRF3 (Pepinh-MyD 0.86 ± 0.25, Pepinh-ctrl 0.56 ± 0.19). These results suggest that the response of DCs to XPa mainly depend on the cGAS-STING pathway and TLRs and that cGAS-STING dominated.

### DCs ultimately undergo apoptosis and pyroptosis

Normally, activated DCs undergo apoptosis within a few days after pathogen invasion in vivo. Consequently, DC apoptosis is crucial for maintaining immune homeostasis. Recently, pyroptosis of DCs was found to be necessary to resist bacterial infection, but excessive pyroptosis can aggravate the inflammatory response.^33^ LPS has been reported to cause pyroptosis in immune cells.^34,35^

**Fig. 5:**
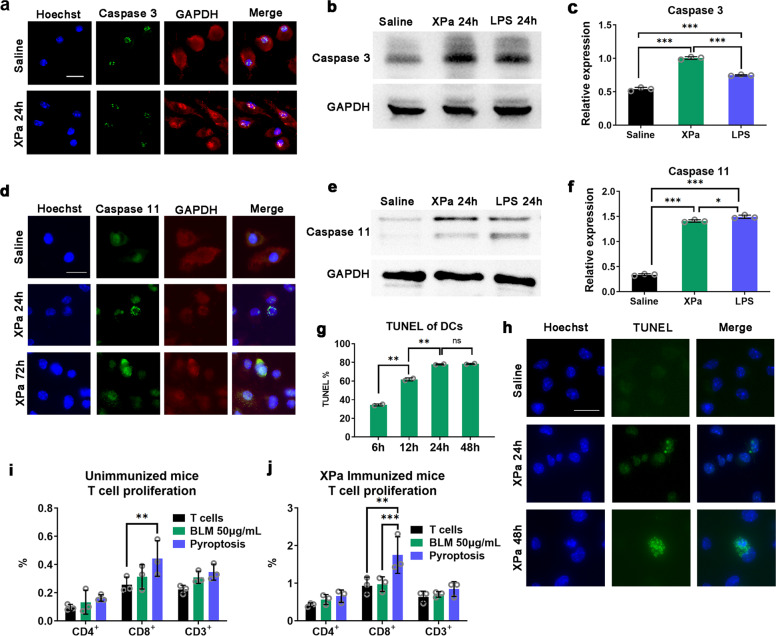
Fate of DCs after XPa immunization in vitro. **a** Immunofluorescence of caspase 3 in XPa (10 MOI)stimulated BMDCs after 24 h. **b** and **c** WB of caspase 3 in RAW264.7 cells treated with saline, LPS (100 ng/mL), and XPa (10 MOI) for 24 h; *n* = 3. **d** Immunofluorescence of caspase 11 in XPa (10 MOI) stimulated BMDCs after 24 h and 72 h. **e** and **f** WB of caspase 11 in RAW264.7 cells treated with saline, LPS (100 ng/mL), and XPa (10 MOI) for 24 h; *n* = 3. **g** TUNEL staining of XPa (10 MOI) stimulated BMDCs after 24 h and 48 h. **h** DCs were stimulated with XPa (10 MOI) for 6, 12, 24, or 48 h and subjected to TUNEL staining (One step TUNEL test kit, Beyotime, C1086). Flow cytometry analysis of TUNEL results; *n* = 2. ns = no significant difference. **i** and **j** DCs were treated with BLM (bleomycin, 50 μg/mL) for 24 h, and pyroptosis was induced (the DCs were aspirated into a syringe, and plunger movements were repeated 10 times). DCs were cocultured with CD3^+^ T cells from the spleens of unimmunized mice or XPa-immunized mice for 72 h. The proliferation of T cells was evaluated using CFSE staining. Scale bars, 20 μm. Data were analyzed via two-way ANOVA. *P < 0.05, **P < 0.01, ***P < 0.001. No letter is present if no significant difference was observed

In our research, both apoptosis and pyroptosis occurred in BMDCs after stimulation. In this study, caspase 3^36^ served as a marker for cell apoptosis and caspase 11^37^ served as a marker for cell pyroptosis. We found that DCs underwent apoptosis (Fig. 5a) and pyroptosis (Fig. 5d) after stimulation by XPa. As a crucial executioner of cell apoptosis, caspase 3 was expressed in almost all DCs in fields, but only a few DCs expressed caspase 11. Caspase 3 was detected in RAW264.7 cells treated with LPS (0.75 ± 0.01) and XPa (1.00 ± 0.02) compared those treated with saline (0.54 ± 0.03) (Fig. 5b, c). Caspase 11 was also detected in RAW264.7 cells treated with LPS (1.49 ± 0.03) and XPa (1.41 ± 0.02) compared with saline (0.34 ± 0.02) (Fig. 5e, f). In addition, TUNEL assay demonstrated positivity during apoptosis and pyroptosis. TUNEL flow cytometry analysis showed a positive result in 34.36 ± 1.10% of cells (Fig. 5g) after 6 h of stimulation, and the positivity peaked (77.85 ± 0.62%) at 24 h, which is consistent with TUNEL staining (Fig. 5h). We concluded that both apoptosis and pyroptosis coregulated the XPa-primed immune response, and apoptosis is the major outcome.

### XPa induced memory T cells

**Fig. 6:**
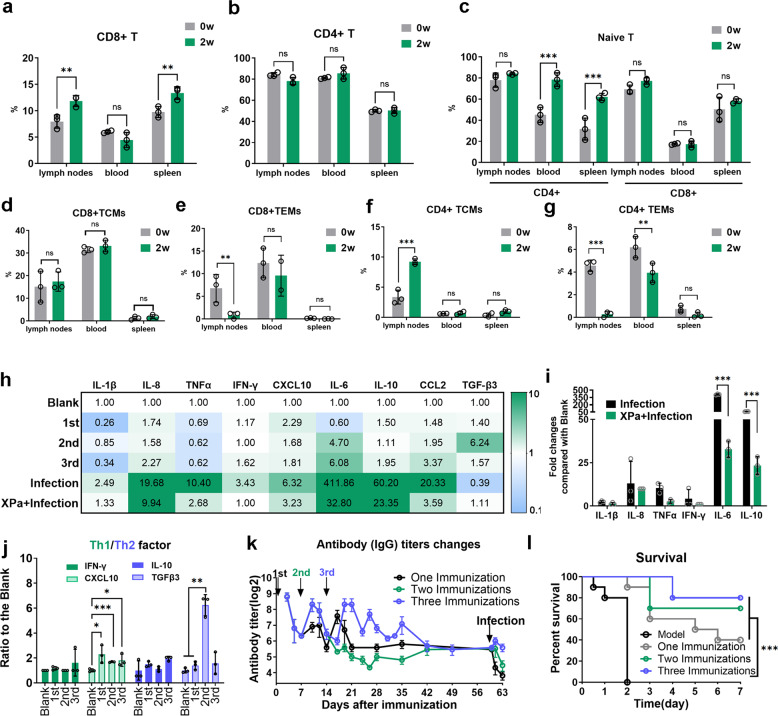
T cells and inflammatory factors in vivo. **a**, **b**, **c** Percentages of CD4^+^, CD8^+^, and naive T cells in the lymph nodes (left-side groin), blood, and spleen of mice after one immunization with XPa subcutaneously on the left side of the groin. *n* = 3. **d**, **e**, **f** and **g** Percentages of central memory T cells (TCMs) and effector-memory T cells (TEMs) among CD4^+^ and CD8^+^ T cells in the lymph nodes (left-side groin), blood, and spleen of mice after one immunization with XPa subcutaneously on the left side of the groin. *w* = week. **h** The factor levels (the fold difference compared with the blank group) in mouse serum after immunizations and infection; *n* = 3. **i** The levels of inflammatory factors, including IL-1β, IL-6, IL-8, TNFα, IFN-γ, and IL-10 in XPa + Infection and Infection mice. **j** Th1/Th2 factor changes during the immunization procedure. **k** The induction of IgG antibodies was monitored after immunization; *n* = 5. **l** The protective efficiency of XPa using different procedures: one immunization 40%, two immunizations 70%, and three immunizations 80%; *n* = 10. Data were analyzed via two-way ANOVA. Survival curves were compared using a log-rank (Mantel–Cox) test (conservative). *P < 0.05, **P < 0.01. No letter is present if no significant difference was observed. **P < 0.01, ***P < 0.001, ns = no significant difference

To explore T-cell types and the change in the immune response to the vaccine, we collected the blood, left-inguinal lymph nodes, and spleens of mice two weeks after one XPa immunization, tested the ratio of CD8^+^ T cells and CD4^+^ T cells (Fig. 6a, b), and analyzed the proportion of naive T cells, effector memory T cells (TEMs) and central memory T cells (TCMs), (Fig. 6c–g) in the CD4^+^ T and CD8^+^ T subsets.^38^ T cells were stained with CD44 and CD62L to identify TEMs (CD44^high+^/CD62L^−^) and TCMs (CD44^high+^/CD62L^+^).

CD8^+^ T cells were increased in the lymph nodes (0 w 7.95 ± 1.25% and 2 w 11.86 ± 1.038%) and spleen (0 w 9.77 ± 0.85% and 2 w 13.37 ± 0.96%) but not in the blood (Fig. 6a), and CD8^+^ TEMs (0w 6.79 ± 3.07% and 2w 0.82 ± 0.70%) in lymph nodes (Fig. 6e) decreased two weeks after XPa immunization, but the number of CD4^+^ T cells did not change (Fig. 6b). The proportion of CD4^+^ naive T cells but not CD8^+^ naive T cells increased in the blood (0 w 45.10 ± 5.79% and 2 w 78.52 ± 5.17%) and spleens (0 w 31.71 ± 8.44% and 2 w 62.20 ± 2.76%) but did not change in the lymph nodes of mice two weeks after XPa immunization (Fig. 6c). The proportion of CD4^+^ TCMs increased in the lymph nodes (0w 3.38 ± 1.15% and 2w 9.24 ± 0.48%) and remained unchanged in blood and spleen (Fig. 6f). The proportion of CD4^+^ TEMs decreased in the lymph nodes (0 w 4.57 ± 0.50% and 2 w 0.27 ± 0.24%) and blood (0 w 6.21 ± 0.76% and 2 w 3.96 ± 0.69%) but did not change in the spleen of mice (Fig. 6g).

To determine whether XPa was active on human T cells, peripheral blood mononuclear cells (PBMCs) were isolated from blood and examined for T cells and the related subtypes after stimulation by XPa (Supplementary Fig. S2). We observed that XPa increased the number of total CD3^+^ T cells (saline 59.45 ± 0.33%, XPa 65.21 ± 0.94%) and CD8^+^ T cells (saline 9.48 ± 0.04%, XPa 11.90 ± 0.34%).

### XPa vaccination induced both Th1 and Th2 cytokine responses in mice and decreased the levels of inflammatory factors in the serum of mice infected with PAO1

The levels of typical Th1 cytokines (IFN-γ and CXCL10) and Th2 cytokines (IL-10 and TGF-β3) were elevated in the serum of mice immunized with XPa (Fig. 6h, j). Compared with the Blank group mice, the level of IFN-γ increased 1.18 ± 0.19 times after the 1^st^ immunization, 1.01 ± 0.00 times after the 2^nd^ immunization and 1.64 ± 1.07 times after the 3^rd^ immunization. The level of CXCL10 increased 2.29 ± 0.72 times after the 1^st^ immunization, 1.68 ± 0.05 times after the 2^nd^ immunization, and 1.81 ± 0.49 times after the 3^rd^ immunization. Compared with the Blank group mice, the level of IL-10 increased 1.49 ± 0.22 times after the 1^st^ immunization, 1.1 ± 0.28 times after the 2^nd^ immunization, and 1.94 ± 0.18 times after the 3^rd^ immunization. The level of TGF-β3 increased 1.39 ± 0.31 times after the 1^st^ immunization, 6.23 ± 0.84 times after the 2^nd^ immunization, and 1.56 ± 0.88 times after the 3^rd^ immunization. Thus, XPa induced Th1 and Th2 responses.

Next, we evaluated the inflammatory factor levels in mouse serum. The results showed that most inflammatory factors, including IL-1β, IL-6, IL-8, and TNF-α, were significantly increased after infection. Greater increases in the levels of IL-1β, IL-6, IL-8, and TNF-α compared with the Blank group were observed in the Infection group than in the XPa + Infection group (XPa + Infection 1.33 ± 0.65 and Infection 2.49 ± 0.57 for IL-1β, XPa + Infection 32.78 ± 6.65 and Infection 411.65 ± 17.52 for IL-6, XPa + Infection 9.95 ± 0.02 and Infection 19.7 ± 8.62 for IL-8, and XPa + Infection 2.67 ± 1.55 and Infection 10.34 ± 2.82 for TNF-α) (Fig. 6i). The specific details of the datasets are presented in Table. S1. This decrease in the change in inflammatory factors suggested a reduced bacterial infection in the XPa + Infection mice compared with the Infection mice. These results support the bactericidal properties and safety profile of XPa in vivo.

Changes in antibody levels in the serum during the study are shown in Fig. 6k. The immunized mice developed robust antibody titers following the third immunization during the studies and maintained high levels from days 21 to 42. Moreover, the mice with three immunizations developed higher antibody titers (ranging from 40 to 80) after infection than those with two immunizations (ranging from 10 to 40) and one immunization (ranging from 10 to 20). The protection effect of different immunization procedures was evaluated by survival rate after challenge (Fig. 6i). We found that multiple immunizations improved survival rate (40%, one immunization; 60%, two immunizations; 80%, three immunizations).

### XPa protected mice in pneumonia models from infection with PAO1 or multidrug-resistant clinical isolate W9

**Fig. 7:**
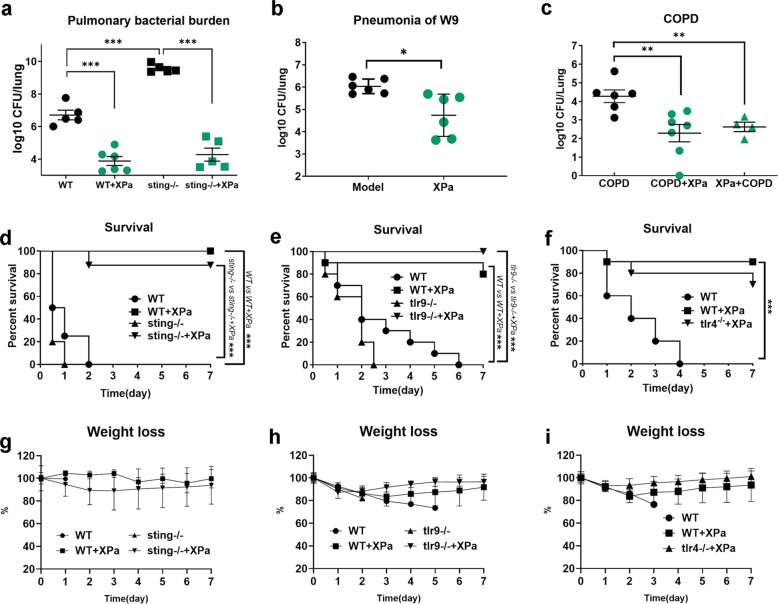
Protective efficiency of vaccine in mice. **a** The pulmonary bacterial burden after challenge with PAO1 in WT mice and *sting*^−/^^*−*^ mice (*n* = 5). **b** The pulmonary bacterial burden in mice after challenge of clinical isolate W9 (*n* = 6). **c** The pulmonary bacterial burden after challenge of PAO1 in COPD model mice vaccinated with XPa. The mice in the COPD + XPa group (*n* = 7) were prepared to establish COPD before immunization. Mice in the XPa + COPD group (*n* = 4) were prepared to establish COPD after immunization. The mice in the COPD group (*n* = 6) were only prepared to establish the COPD model. See “Materials and methods” for detailed experimental processes. **d** Survival curve of the mice after challenge with PAO1 (*n* = 8). **e** and **f** Survival curves of *tlr4*^*−/−*^, *tlr9*^*−/−*^ mice after challenge of PAO1 and of *tlr4*^*−/−*^, *tlr9*^*−/−*^ mice immunized with XPa (*n* = 5). **g**, **h** and **i** Bodyweight curves throughout the experiment. Data were analyzed using unpaired *t*-test. Survival curves were compared using a log-rank (Mantel–Cox) test (conservative). *P < 0.05, **P < 0.01. No letter is present if no significant difference was observed

XPa demonstrated positive protective results in a murine model of acute bacterial pneumonia caused by PAO1. A lower bacterial burden in the lungs was found in the immunized group (log10 3.88 ± 0.67) than in the WT group (log10 6.71 ± 0.66) after challenge with PAO1 (Fig. 7a). During the experiment, no differences were observed in the weights of the XPa-treated animals, except in the WT mice (the WT mice were all dead by day 2) (Fig. 7g). Overall, our data indicate that XPa protects mice from PAO1 challenge.

The cGAS-STING pathway was activated in vitro, but this activation was not clear in vivo. To this end, *sting*^*−/−*^ mice were used in additional experiments. The *sting*^*−/−*^ mice showed a significantly higher bacterial burden (log10 9.56 ± 0.25) in the lungs than the WT mice (log10 6.71 ± 0.66) (*P* < 0.001) and had a shorter survival time. The *sting*^*−/−*^+XPa mice exhibited a lower bacterial burden (log10 4.28 ± 0.90) than the *sting*^*−/−*^ mice (log10 9.56 ± 0.25) (*P* < 0.001) (Fig. 6a). We also performed the same assay using *tlr4*^*−/−*^ and *tlr9*^*−/−*^ mice (Fig. 7e, f). Collectively, the results indicate that the cGAS-STING pathway mediates the immune response elicited by XPa in mice.

*P. aeruginosa* is very difficult to prevent and treat due to its multidrug resistance. Thus, we repeated the experiments using the clinically isolated multidrug-resistant *P. aeruginosa* W9 (serotype O20) (Fig. 7c). Lower-lung W9 load was found in mice immunized (log10 4.74 ± 0.95) with XPa than those in unimmunized mice (log10 6.03 ± 0.33) (*P* < 0.01). This result suggests that XPa was able to overcome the drug resistance of bacterial pathogens and protected mice from challenge with the heterologous serotype W9 (PAO1 was serotype O2/O5).

### COPD mice immunized with XPa could resist PAO1 infection

*P. aeruginosa* is detectable in the airways of approximately 25–50% of clinically stable COPD patients,^39^ and most COPD patients have sporadic and intermittent airway infection with *P. aeruginosa*, which is repeatedly cleared.^40^ We further measured the protective efficacy of XPa in a COPD mouse model infected with PAO1 (Fig. 7b). The bacterial load in the lungs of mice in the XPa + COPD (log10 2.62 ± 0.51) and COPD + XPa (log10 2.29 ± 1.23) groups decreased (**P < 0.01) compared to that in the COPD group (log10 4.27 ± 0.84), which hinted at the potential for clinical application of XPa.

## Discussion

Repeated infections and escape from immune surveillance are the two primary difficulties in the clinical treatment of *P. aeruginosa* infection, and strategies to regulate the immune response and produce immunological memory are needed. Here, we report an inactivated whole-cell vaccine against *P.aeruginosa* named XPa. XPa showed good protection in a mouse acute-pneumonia model induced by either PAO1 or the clinical isolate strain W9 (MDR strain), even in a COPD model of infection with PAO1.

In contrast to traditional inactivated vaccines, XPa was prepared by X-ray irradiation, which has been reported to damage DNA.^31^ This explains why we detected a high quantity of small-fragment nucleic acids in the XPa supernatant. Short DNAs of ~20 bp have been reported to bind to cGAS, but longer dsDNAs of >45 bp can form more stable ladder-like networks of cGAS dimers.^41^ Studies have also verified that RNA: DNA hybrids can activate the cGAS-STING pathway by binding to cGAS.^42^ In addition, 8-OHG was reported to activate the cGAS-STING pathway in immune cells.^43,44^ Therefore, the nucleic acids in XPa activated the cGAS-STING pathway. This is consistent with our in vitro experimental results.

Phagocytosis, antigen processing, and subsequent antigen presentation to T cells are important immunological functions of DCs. Immature DCs exhibit a high endocytic rate that enables efficient antigen uptake, upon stimulation, DCs transition to a mature cell type characterized by low phagocytic capacity. LPS and gum arabic have been reported to decrease the phagocytic capacity of mouse BMDCs.^45^ Our study suggested that XPa containing LPS could decrease the phagocytic capacity of DCs as shown by FITC– dextran uptake. Mature DCs are pivotal for T-cell activation and polarization.^46^ DC maturation induced by XPa was associated with specific activation of CD8^+^ T cells.

Considering the comprehensive immune response to the whole-cell vaccine, TLRs (MyD88 dependent) and the cGAS-STING pathway (MyD88 independent) were compared in this study. MyD88 is the intracellular segment structure in TLR-family receptors and plays a key role in activation of the TLR family, and MyD88 was also reported to affect TBK1 activation in TLR4 signaling.^47^ In the present study, we found that *sicgas* reduced the total TBK1 (pTBK1 + TBK1) and pIRF3/IRF3 ratios. However, a MyD88 inhibitor affected the pTBK1/TBK1 ratio but not the pIRF3/IRF3 ratio or total TBK1, and the Pep-inh MyD and the Pep-inh ctrl did not show significant differences. In conclusion, the cGAS-STING pathway is the main effector in DCs stimulated by XPa.

Due to the important role of DCs in the immune response, we studied how XPa influences the functions of DCs. One key point of interest was DC fate. DC fate plays an important role in the immune response, and dysregulation of DC homeostasis may be a broad strategy of pathogens to evade immunity and facilitate travel to distant sites within the host. An appropriate vaccine should induce a quick, wild but not strong immune response.^48^ When damaged by pyroptosis, DCs rupture and release cellular contents and inflammatory factors, such as TNF-α and IL-1β, aggravating the inflammatory response. Thus, apoptosis is generally anti-inflammatory and pyroptosis induces inflammation. They both regulated the intensity of immune responses to XPa in mice, and no severe inflammatory responses were observed in immunized mice (the levels of the inflammatory factors IL-6, TNF-α and IL-8 were lower than those of Infection group mice). Therefore, we concluded that the predominant mode of DC death in XPa-primed immune response was apoptosis.

We also studied T-cell proliferation when T cells were cocultured with apoptotic or pyroptotic BMDCs (Fig. 5i, j). Bleomycin^49^ (50 μg/mL, 24 h) was used to induce apoptosis of DCs and an insulin syringe was used to disrupt the cells to produce cell fragments, which simulated the cell debris produced by pyroptosis. The experimental procedures were the same as those described in the section on T-cell proliferation. In unimmunized mice, CD8^+^ T cells significantly proliferated (0.44 ± 0.13%, **P < 0.01) when cocultured with pyroptotic DCs, and in XPa-immunized mice, CD8^+^ T cells significantly proliferated (1.75 ± 0.49%, ***P < 0.001) when cocultured with pyroptotic DCs, but CD4^+^ T cells (unimmunized mice 0.31 ± 0.09% and immunized mice 0.98 ± 0.20%) did not show significant differences with the T-cell group. We detected an increase in CD8^+^ T cells in the lymph nodes, which might be associated with DC pyroptosis.

In addition, immunological memory is critical for the function of any vaccine. Therefore, memory T cells were detected in the study. Categorization of memory T cells into TCM and TEM subsets has already been proposed. TCMs retain the capacity to recirculate throughout lymphoid tissue, while TEM cells migrate to nonlymphoid sites and are programmed to induce inflammatory responses in situ upon Ag re-exposure.^50^ We found an expansion of CD4^+^ TCMs and depletion of CD4^+^ and CD8^+^ TEM cells in mouse lymph nodes 2 weeks after XPa injection. In human peripheral lymphocytes, we found expansion of CD8^+^ cells and CD4^+^ TEMs after XPa treatment (Supplementary Fig. S3).

XPa helped to maintain immune balance and reduce infection. A balance of Th1/Th2 cells in the body is defined as immune balance. In natural immunity, the Th1 response externalizes the infection and provides permanent immunity. Th2 cells respond with antibodies and ensure immunity, but Th1 and Th2 cells work in tandem.^51^ Most vaccines using adjuvants promote a failure in immune balance by making Th2-type cells dominant,^52^ bypassing Th1 cells, which puts the immune system into a state of confusion. Our vaccine induced both Th1 and Th2 cytokines, which could be explained by the ability of XPa to simulate natural infection conditions and its weak infection and vaccine properties. Infection in mice has been associated with overproduction of proinflammatory cytokines, which leads to cytokine storms and systemic inflammatory-response syndrome. These factors are the major inflammatory mediators associated with bacterial infection, and XPa immunization significantly decreased these factors during infection. These results demonstrate the bacterial-clearance ability of XPa.

Notably, the proinflammatory factor IL-6 and anti-inflammatory factor IL-10 were both increased during infection. In 1997, Roger Bone proposed compensatory anti-inflammatory response syndrome (CARS) to qualify the consequences of the counterregulatory mechanisms initiated to limit the overzealous inflammatory process in patients with infectious (sepsis) or noninfectious systemic inflammatory-response syndrome (SIRS).^53^ In the present study, PAO1 pneumonia may lead to systemic bacterial distribution in mice. Systemic distribution of bacteria plays an essential role in the induction of sepsis, SIRS, and CARS.^54^ While SIRS is associated with massive secretion of proinflammatory cytokines, including IL-6, CARS is linked to production of the regulatory cytokine IL-10.^54^ Moreover, CARS and SIRS are not temporally distinct phases, and may coexist and overlap within the same patient.^55^ In this study, mice with PAO1 infection show the upregulated IL-6 and IL-10 levels.

XPa facilitated tissue repair of the body. The TGF-β pathway is central to fibrosis progression in multiple organs.^56^ Specifically, TGF-β1 and TGF-β2 may promote scar formation, whereas TGF-β3 may reduce scarring. Pulmonary fibrosis is one outcome of repeated inflammation. COPD exacerbations have been associated with increased markers of inflammation acts in the airway,^57^ and inflammation acted as an important driver in the development of pulmonary fibrosis.^58^ In our study, TGF-β3 levels (6.23 ± 0.84) in mice of the 2nd Immunization group increased significantly, while the TGF-β1 and TGF-β2 levels did not change much (data not shown). XPa exerted inhibitory effects on pulmonary fibrosis in vivo.

CCL2 is best known for its role in regulating macrophage recruitment and M2 polarization during inflammation,^59^ and M2 macrophages generate high levels of the anti-inflammatory cytokine IL-10.^60^ M2 macrophages are anti-inflammatory and have been reported to facilitate wound repair.^61^ We detected increased levels of CCL2 throughout the vaccination period, which was consistent with the variation trends in IL-10. These results hinted at the possibility that XPa inhibits inflammation and helps the body repair tissue.

In general, the second immunization was used as a boost. For example, antibody levels tended to be expressed at a higher level after the second immunization.^62–65^ However, compared with levels after the first dose, we discovered that many factors were not higher following a second dose. This result suggests that the immune response was reduced after the second immunization, which is similar to the findings of previous studies.^66,67^ This reduction in the acute-phase response to the second vaccination could be explained by the fact that the mice were partially immune on the latter occasion, they were immunologically naive at the primary vaccination but not at the time of the secondary vaccination.^67^

Further, this study further revealed the mechanism of the X-ray-inactivated whole-cell vaccine XPa against *P. aeruginosa* infection. As a vaccine with nucleic acids, an in vitro experiment was revealed that primarily acts through the cGAS-STING pathway. In the immune response, DCs mature after XPa stimulation, successfully present antigens to T cells, induce T-cell activation and proliferation, and ultimately undergo apoptosis and pyroptosis. The vaccination generated memory-immune responses that protected mice from *P. aeruginosa* challenge and decreased the levels of inflammatory factors in the serum of infected mice. Overall, in this study, a new mechanism of an irradiated inactive whole-cell vaccine against *P. aeruginosa* infection was discovered.

## Methods and materials

### Cell lines, strains, and animals

RAW264.7 cells were cultured in RPMI 1640 medium (HyClone) supplemented with 10% fetal bovine serum (FBS, Gibco).

The bacterial strains PAO1 and PAO1-GFP were preserved in our laboratory. The PAO1-GFP strain utilized in the study was constructed with pBK-mini-Tn7-rrnBP1-gfpAGA using the mini-Tn7 system as previously described.^68^ W9 is a clinically isolated multidrug-resistant (resistant to β-lactams, cephalosporins, tetracycline, sulfamethoxazole, nitrofurantoin, aztreonam, imipenem, and meropenem) *P. aeruginosa* strain, and according to International Antigenic Typing Schema (IATS), the serotype of W9 is O20.

Female C57BL/6 mice were purchased from Beijing HFK Bioscience Co. Ltd. *sting*^*−/−*^(017537) C57BL/6 mice were purchased from the Jackson Laboratory. *tlr4*^*−/−*^ C57BL/6 mice were purchased from GemPharmatech Co. Ltd. (Nanjing, China). *tlr9*^*−/−*^ C57BL/6 mice were provided by the Institute of Laboratory Animal Resources (Beijing, China) and were originally generated by Oriental BioService Inc. (Kyoto, Japan) through homologous recombination. The mice were 6–8 weeks old at the time of the first immunization.

All animal experiments were approved by the Institutional Animal Care and Use Committee of Sichuan University. All mice were maintained under specific pathogen-free conditions, and all efforts were made to minimize mouse suffering. All animals in this study were treated according to the guidelines of Sichuan University and with the approval of the Ethics Committee of Sichuan University.

### Preparation of XPa

Exponentially growing *P. aeruginosa* PAO1 cells in 6 L tryptic soy broth (TSB) medium were harvested by centrifugation (5,316 g, 20 min). The cells were washed once with 30 mL of 0.9% sodium chloride (saline) in a sterile 50-mL centrifuge tube and adjusted to an OD_600_ of 50 with saline. OD_600_ that represents the ultraviolet-absorbance value of the bacterial suspensions was at 600 nm. After PAO1 was collected in saline, 300 μL of the bacterial suspensions were diluted 10–100-fold and optical densities were measured at 600 nm (OD_600_) in a spectrophotometer (S1200 Diode Array Spectrophotometer, DENVILLE SCIENRIFIC INC.). The OD_600_ of the concentrated PAO1 suspension was adjusted to 50 with saline according to the OD_600_ of the diluted PAO1 suspension. For PAO1, OD_600_ = 50 means that the bacterial concentration is approximately 1–2 × 10^10^ CFU/mL.

The adjusted PAO1 suspension was divided into two parts: one without irradiation was used as a control (PAO1), and the other was exposed to X-ray irradiation at a dose rate of 7.086 Gy/min using an RS2000 Biological X-ray irradiator (Rad Source Technologies, FL, USA) at 160 kV/25 mA. The irradiation procedure was 7.086 Gy/min × 20 min/times × 7 times.

### Detection of nucleic acids and 8-OHG content

A Nanodrop 2000 spectrophotometer (Thermo Fisher Scientific, United States) was used throughout the study to quantify nucleic acids.

The 8-OHG levels in XPa were quantified using a DNA Damage 8OHdG ELISA Kit (SKT-120; StressMarq Bioscience, Victoria, Canada) in accordance with the manufacturer’s instructions.

### Transmission electron microscopy

For formaldehyde-treated PAO1, PAO1 was treated with 1% formaldehyde at room temperature for 48 min and then washed with saline. PAO1 without treatment was used as a control.

For the bacterial cells, the samples were fixed with 2.5% glutaraldehyde/PBS for 2 h at 4 °C. The prepared bacterial samples were sent to the Center of Forecasting and Analysis of Sichuan University (Sichuan, People’s Republic of China) for transmission electron microscopy (Tecnai G2 F20 S-TWIN, FEI) imaging.

### Confocal microscopy of PAO1-GFP

An exponentially growing *P. aeruginosa* PAO1-GFP culture in TSB medium at 37 °C was harvested by centrifugation, washed in sodium chloride solution, and resuspended to an OD_600_ of 50 in sodium chloride solution. PAO1 without treatment was used as a control. XPa and PAO1 were stained with PI (50 ng/mL) for 5 min and observed on a 1.2% agarose pad using N-STORM&A1 microscope (Nikon) at 488 nm and 594 nm. XPa + DNase I samples were treated with DNase I (10 μg/mL) at 37 °C for 1 h, and then, the staining step was conducted. All samples were transferred to a thin agar pad on a microscope slide and imaged with the N-STORM&A1 microscope system (Nikon).

### Analysis of nucleic acid size distribution

The size distributions of the nucleic acids in the supernatant of PAO1 and XPa were assessed via agarose gel electrophoresis and 4–20% polyacrylamide TBE gel electrophoresis. The samples were centrifuged at 17,000 × *g*, and the supernatants were collected and filtered with a 0.22-μm filter. The obtained solutions were separated via gel electrophoresis (1% agarose) in 1 × TBE buffer at 90 V for 15 min. The obtained solutions were separated in 4–20% polyacrylamide TBE gel (Invitrogen, EC62255BOX) in 1 × TBE buffer for 30 min. The gel was stained with SYBR Gold Nucleic Acid Gel Stain. Images were detected with an enhanced chemiluminescence system (iBright CL1000, Thermo Fisher). The agarose electrophoresis image was inverted, the gray peaks were analyzed using ImageJ software, and a curve was drawn to analyze the nucleic acids.

### DC isolation, culture, and stimulation

First, bone marrow (BM) cells were flushed from the femurs and tibiae of mice with RPMI 1640 medium (HyClone). The suspension was filtered through a 70-μm mesh, transferred to a sterile 50-mL centrifuge tube, and centrifuged for 5 min at 227 × *g*. Red blood cells (RBCs) were lysed using RBC lysis buffer on ice for 10 min. The cells were washed and resuspended in fresh medium. Fresh medium contained 10% heat-inactivated FBS (Gibco), 50 mM 2-mercaptoethanol (Sigma), and 20 ng/mL recombinant murine GM-CSF (315-03, PeproTech, Rocky Hill, NJ, USA). Half of the medium was changed every two days. On day 7, the nonadherent cells were used in experiments as immature BMDCs.

For DC maturation: After DC collection and counting, the cells were seeded into 6-well plates at 1 × 10^6^ cells per well. The DCs were treated with saline, LPS (100 ng/mL; Sigma L9143), XPa at an MOI of 10, XPa sup (XPa was centrifuged at 17,000 × *g*/min × 10 min and filtered through a 0.22-μm filter to obtain XPa sup. XPa sup was added at the same volume as XPa), and XPa + DNase I (XPa was treated with DNase I at 37 °C for 1 h) at an MOI of 10 for 24 h. The DCs were collected for flow cytometry.

For FITC–dextran uptake: After DC collection and counting, the cells were seeded into 6-well plates at 1 × 10^6^ cells per well. The DCs were treated with saline, LPS (100 ng/mL; Sigma), XPa at an MOI of 10, XPa sup (XPa was centrifuged at 17,000 × *g*/min × 10 min and filtered through a 0.22-μm filter to obtain the XPa sup. XPa sup was added with the same volume of XPa), and XPa + DNase I (XPa was treated with DNase I at 37 °C for 1 h) at an MOI of 10 for 24 h. Then, the FITC–dextran was added for 24 h at a final concentration of 10 µg/mL. After washing with PBS to remove the free FITC–dextran, the cells were stained with an anti-CD11c-PE antibody in the dark for 30 min at room temperature. Finally, the cells were washed in PBS twice and analyzed with a Novocyto flow cytometer (Agilent Biosciences). A portion of the cells were placed in another 6-well plate and photographed with a Nikon Eclipse Ti-inverted microscope (Nikon, Tokyo, Japan).

For western blotting: After DC collection and counting, the cells were seeded into 6-well plates at 1 × 10^6^ cells per well. Then, the DCs were treated with saline or XPa at an MOI of 10.

### Flow cytometry

Cells were washed with PBS and stained with anti-MHC class II-APC (BioLegend, 107613), anti-CD80-PE-Cy5 (BioLegend, 104712), anti-CD86-APC-Cy7 (BioLegend, 105030), and anti-CD11c-FITC (BioLegend, 117306) antibodies at room temperature (RT) for 30 min in the dark. Then, the samples were washed and detected with a Novocyto flow cytometer (Agilent Biosciences). Isotype controls were set as the negative controls.

The mice were sacrificed, and blood, left-inguinal lymph nodes, and spleens were harvested. The lymph nodes were stripped and torn in PBS, spleens were gently pressed through cell strainers (70 μm) with the plunger of a syringe into PBS in a Petri dish, and the suspensions were treated with RBC lysis buffer on ice for 10 min and washed with PBS; blood was treated with RBC lysis buffer for 10 min and washed with PBS. The samples were stained with anti-CD3-APC-Cy7 (Biolegend, 100222), anti-CD4-PE (Biolegend, 100407), anti-CD8a-PE-Cy7 (Biolegend, 100722), anti-CD44-APC (Biolegend, 103012), and anti-CD62L-Fluor 488 (Biolegend, 104420) antibodies for 30 min at RT in the dark. Isotype controls were set as the negative controls. Samples were tested and analyzed using the Novocyto flow cytometry.

Human PBMCs from human peripheral blood were stained with anti-CD3-PE 7 (Biolegend, 300308), anti-CD4-APC (Biolegend, 317416), anti-CD8a-PE-Cy7 (Biolegend, 344750), anti-CD45RO-PerCP (Biolegend, 304251), and anti-CD62L-PE (Biolegend, 304805) antibodies after the treatment and analyzed using the Novocyto flow cytometer. Isotype controls were set as the negative controls.

### Proliferation of T cells

Spleens were harvested from female C57BL/6 mice (4–6 weeks old) that were previously immunized with XPa one week before being sterilized. Cell suspensions were generated by filtration through a 70-μm nylon-mesh filter (BD Biosciences), and lymphocytes were enriched via specific separation medium and density-gradient centrifugation and isolated with an EasySep Mouse T cell Isolation Kit (19851, STEMCELL Technologies). Then, T cells were stained with 1 mM carboxyfluorescein (CFSE, 423801, Biolegend Inc., San Diego, CA, USA), and CFSE-stained T cells were cocultured with DCs treated with saline, LPS (100 ng/mL; Sigma L9143), XPa (10 MOI), XPa sup (same volume of XPa), XPa + DNase I (10 MOI), or CD3/CD28 (Dynabeads Mouse T-Activator CD3/CD28, Gibco, 11452D) at a DC:T-cell ratio of 1:10. After 72 h of coculture, cells were collected for flow cytometry analysis, and culture supernatants were collected for cytokine IFN-γ assays.

### Immunofluorescence

For immunofluorescence assays, cells were plated into 6-well plates and stimulated with XPa at an MOI of 10 for 24 h. Then the cells were fixed with 4% paraformaldehyde in phosphate-buffered saline (PBS). After permeabilization with 0.1% Triton X-100 (Sigma) in PBS, the cells were blocked in a solution containing 2.5% BSA (Sigma) and 2.5% goat serum for 1 h and then stained with specific primary antibodies (CST) (1:200) followed by secondary antibodies (1:200) (Alexa Fluor Plus 488 goat anti-rabbit and Alexa Fluor Plus 594 goat anti-mouse; Invitrogen, Thermo Fisher Scientific). Finally, the cells were stained with DAPI, and the slides were sealed with antifluorescence-quenching sealing tablets. Stochastic optical reconstruction microscopy (STORM) and total internal reflectance fluorescence (TIRF) images were obtained on the N-STORM&A1 microscope system (Nikon).

### siRNA transfection

DCs were seeded into a 6-well plate at 10^6^ cells/well. Then, the cells were transfected with 110 pmol/well nontargeting control small-interfering RNA (*sictrl*) or cGAS-specific siRNA (*sigas*) (sense: GGAUUGAGCUACAAGAAUATT; antisense: UAUUCUUGUAGCUCAAUCCTT) (GenePharma Inc, Shanghai, China), using a transfection reagent (Polyplus Transfection, Illkirch, France). After 48 h of transfection, the knockdown efficiency was confirmed by western blotting.

### MyD88 inhibition

BMDCs were seeded into a 6-well plate at 10^6^ cells/well. Then, the cells were treated with Pepinh-MyD or Pepinh-ctrl (InvivoGen, tlrl-pimyd) (50 μg/mL) for 6 h.

### Western blotting

Cells were collected by centrifugation and lysed in sample buffer (10 mL of 1 M Tris 6.8, 4 g of SDS, 100 mg of BroBlue, 20 mL of glycerol, 10 mL of β-Me, and ddH_2_O to 200 mL) with phosphatase-inhibitor cocktail and complete protease-inhibitor cocktail for 20 min on ice and boiled for 10 min. The lysate was centrifuged at 17,000 × *g* for 15 min at 4 °C to remove precipitates.

Proteins were separated via SDS-PAGE and transferred to PVDF membranes, which were washed in Tris-buffered saline with 0.1% Tween-20 (TBST) and blocked with 5% skim milk. Next, the membranes were incubated with primary antibodies overnight at 4 °C, washed with TBST, and then incubated with horseradish peroxidase (HRP)-labeled secondary antibodies. Finally, an enhanced chemiluminescence system (iBright CL1000, Thermo Fisher) was used to detect proteins.

RAW264.7 cells in logarithmic growth phase were collected and seeded into a 6-well plate at 10^6^ cells/well. After 24 h, the cells were treated with saline, LPS (100 ng/mL), and XPa at an MOI of 10 for 24 h and collected for western blotting.

### HEK-293T cells stably expressing hSTING-GFP and IFN-β-firefly luciferase reporter detection

Tmem173-gfp lentiviruses were produced by GeneChem Inc. A stable hSTING-293T cell line was generated by infection with the tmem173-gfp lentivirus followed by selection with puromycin (0.5 μg/mL). 293T-STING-GFP cells were analyzed via flow cytometry (BD FACS Aria II) on the 488-nm channel. The expression of two genes (*tmem173* and *egfp*) in the cells was assessed by quantitative PCR (qPCR).

An IFN-β-firefly luciferase reporter plasmid was obtained from HANBIO Inc. pGL3 was the vector and contained a luciferase gene. The reporter plasmid was constructed by inserting an IFN-β promoter into pGL3 before the luciferase gene. 293T-STING-GFP cells were seeded in a 96-well plate and transfected with the IFN-β-firefly luciferase reporter plasmid using Polyplus (jetPRIME) for 24–48 h. Then the saline, XPa (10 MOI), and cGAMP (16.5 μg/mL) were added to the wells. Luciferase activity was detected with a Dual Luciferase Reporter Gene Assay Kit (Beyotime, RG027).

### cDNA preparation and quantitative PCR

Cells were harvested and resuspended in 1 mL of TRIzol (Life Technologies, # 15596018) and the mixture was frozen at −80 °C, until use. For RNA extraction, 0.2 mL of chloroform was added after thawing, and samples were thoroughly mixed. Samples were incubated at RT for 2 min before centrifugation (15 min at 12000×g and 4 °C). The aqueous phase was removed and mixed with an equal volume of isopropanol. The RNA pellet was collected by centrifugation and washed with 70% ethyl alcohol. Then the pellet was dissolved in 50 μL of nuclease-free water. RNA from each sample was used to generate cDNA using a HiFiScript gDNA Removal cDNA Synthesis Kit (CWBIO, # CW2582M) according to the manufacturer’s instructions.

Two microliters of each cDNA reaction was used for quantitative PCR with FastSYBR Mixture (CWBIO, CW0955M CW2621M CW2622M), carried out on a LightCycler 96 (Roche). The hSTING (*tmem173*) primer sequences were ATGAGGCGGCAGTTGTT and GGAGGATAGGGTTGAGCAG, the EGFP primer sequences were GAAGAACGGCATCAAGGTG and CGGACTGGGTGCTTAGGTAG, and the readings were normalized to GAPDH expression using the primers AATCCCATCACCATCTTCCA and TGGACTCCACGACGTACTCA. The following temperature program was used: 5 min at 95 °C; 40 cycles of 10 s at 95 °C, 20 s at 52 °C, and 20 s at 72 °C.

### Immunization and challenge

Mice were immunized subcutaneously three times with 1 × 10^7^ CFU XPa in 50 μL in the left-inguinal region, with a one-week interval between each immunization. Seven days after the last immunization, mice were challenged with intratracheal administration of 50 μL of approximately 1 × 10^7^ CFU/mL PAO1 or 1 × 10^9^ CFU/mL W9. Twenty-four hours after the challenge, the lungs of mice were harvested for enumeration of colonies.

### Elastase-induced COPD mouse model and the challenge

An elastase-induced COPD mouse model was established previously.^69^ Briefly, C57BL/6 mice were anesthetized with sodium pentobarbital (80–100 mg/kg intraperitoneal) and then, the mice were treated with a single application of 0.44 U/mouse porcine pancreatic elastase (E1250, Sigma Aldrich) in 25 μL of PBS by airway perfusion. The mice in the COPD + XPa group were treated with elastase two days before the first injection of the immunization program (days 0, 3, and 7), and the PAO1 challenge (5 × 10^5^ CFU/mouse) was carried out via airway perfusion one week after the third immunization. The mice in the XPa + COPD group were treated with elastase 10 days before the challenge, and the first injection of the immunization program (days 0, 3, and 7) was conducted five days before the elastase treatment.

### Cytokine detection

Blood was collected from the orbital vein of mice using heparin-coated capillary tubes 24 h after immunization and infection, and serum was obtained after coagulation and collected via centrifugation. The cytokines secreted in the serum of immunized mice after stimulation in vivo were detected using a cytokine/chemokine magnetic bead panel (MCYTOMAG-70k-11 and TGFBMAG-64k-03, MILLIPLEX MAP).

### Statistical analysis

All data were analyzed using GraphPad Prism software (GraphPad, San Diego, CA). Data were analyzed using one-way or two-way ANOVA (multiple groups) and unpaired *t*-tests. *P < 0.05, **P < 0.01, and ***P < 0.001 were considered statistically significant in all experiments. All values are presented as the mean ± SD.

## Supplementary information

supplementary materials

Movie S1

Movie S2

Movie S3

Movie S4

## Data Availability

The additional data collected during this study are available from the corresponding author upon reasonable request.
