# Supplementary material for: A novel inactivated whole-cell Pseudomonas aeruginosa vaccine that acts through the cGAS-STING pathway
Source: Signal Transduct Target Ther. 2021 Oct 1;6:353. doi: 10.1038/s41392-021-00752-8 (PMC8484301; doi:10.1038/s41392-021-00752-8)
Supplement: Supplementary file 1 — supplementary materials [file 41392_2021_752_MOESM1_ESM.docx]

Supplementary Materials for

A novel inactivated whole-cell *Pseudomonas aeruginosa* vaccine that acts through the cGAS-STING pathway

Cuicui Ma, Xiao Ma, Boguang Jiang, Hailong Pan, Xueyuan Liao, Li Zhang, Wenfang Li, Yingjie Luo, Zhixue Shen, Xingjun Cheng, Mao Lian, Xiawei Wei, Zhenling Wang*

Correspondence to: wangzhenling@scu.edu.cn

**This PDF file includes:**

Figures. S1 to S2

Table S1

Captions for Movies S1 to S4

**Other Supplementary Materials for this manuscript include the following:**

Movies S1 to S4


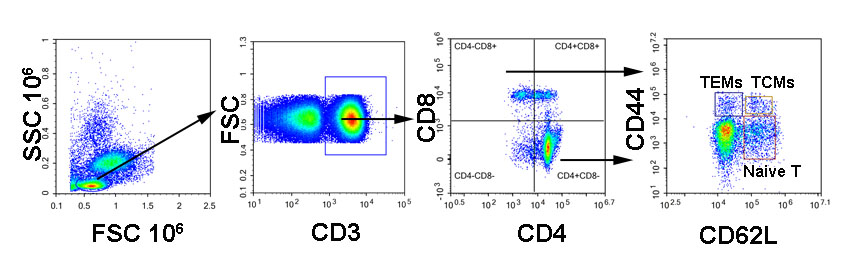


Figure. S1.

Gating strategy for flow cytometry and analysis of T cells in mouse lymph nodes, blood and spleen.


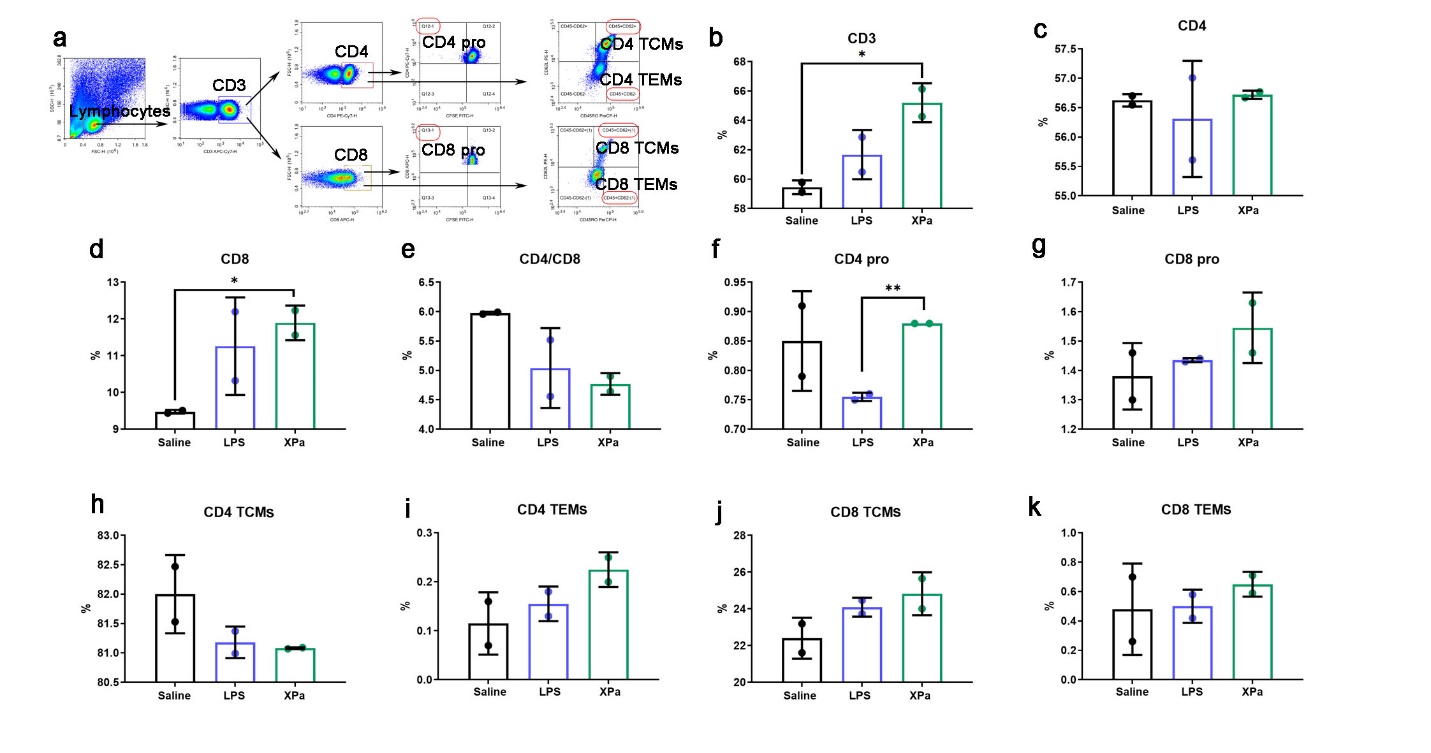


Figure. S2.

Subtypes of T cells among PBMCs after exposure to XPa. **a** Gating strategy for flow cytometry and analysis of T cells. **b** Proportions of T cells. **c** Proportions of CD4^+^ T cells. **d** Proportions of CD8^+^ T cells. **e** Ratio of CD4^+^/CD8^+^ T cells. **f** Proliferation of CD4^+^ T cells. **g** Proliferation of CD4^+^ T cells. Pro= proliferation. **h** Proportions of CD4 TCMs. **i** Proportions of CD4 TEMs. **j** Proportions of CD8 TCMs. **k** Proportions of CD8 TEMs. First, PBMCs were isolated from human peripheral blood (The blood samples were obtained from healthy volunteers with no history of *P.aeruginosa* infection.) using lymphocyte separation buffer and labeled with CFSE. Second, PBMCs were seeded in 6-well plates (10^6^/well) and stimulated with LPS (100 ng/mL), and XPa (10 MOI) for 72 h, Saline as negative control. Finally, the T cells were detected by flow cytometry. Data were analyzed using an unpaired t-test. *P<0.05, **P<0.01. No letter is present if no significant difference was observed.

Table S1.

Inflammatory factor levels (mean±SD, fold changes compared with the blank group) in mouse serum after immunizations and infection; n=3. Data were analyzed using an unpaired t-test. The numbers were compared with the Blank values. *P<0.05, **P<0.01, ***P<0.001.

|  | IL-1β | IL-8 | TNFα | IFN-γ | CXCL10 | IL-6 | IL-10 | CCL2 | TGFβ3 |
| --- | --- | --- | --- | --- | --- | --- | --- | --- | --- |
| Blank | 1.00±0.74 | 1.00±1.18 | 0.99±0.42 | 1.01±0.00 | 1.00±0.12 | 1.00±0.34 | 1.00±0.78 | 1.00±0.55 | 1.00±0.19 |
| 1st | 0.26±0.01 | 1.74±0.62 | 0.68±0.12 | 1.18±0.19 | 2.29±0.72** | 0.60±0.28 | 1.49±0.22 | 1.48±0.55 | 1.39±0.31 |
| 2nd | 0.85±0.84 | 1.58±1.00 | 0.61±0.00 | 1.01±0.00 | 1.68±0.05 | 4.70±4.50 | 1.1±0.28 | 1.95±0.49 | 6.23±0.84  ** |
| 3rd | 0.34±0.10 | 2.27±0.97 | 0.61±0.00 | 1.64±1.07 | 1.81±0.49 | 6.07±5.27 | 1.94±0.18 | 3.37±2.23 | 1.56±0.88 |
| Infection | 2.49±0.57 | 19.7±8.62 | 10.34±2.82* | 3.47±4.2 | 6.32±2.09*** | 411.65±17.52  *** | 59.92±2.62  *** | 20.35±4.51  ** | 0.39±0.02 |
| XPa+  Infection | 1.33±0.65 | 9.95±0.02* | 2.67±1.55 | 1.01±0.00 | 3.23±0.16* | 32.78±6.65  ** | 23.25±7.20  * | 3.59±0.00  * | 1.11±0.91  * |

Movie S1.

Snapshots of the resulting 3D reconstruction movie of BMDCs.

Movie S2.

Snapshots of the resulting 3D reconstruction movie of stimulated with XPa for 3 h.

Movie S3.

3D reconstruction movies of 239T-GFP cells labeled with ER-Tracker Red (Thermo Fisher, E34250).

Movie S4.

3D reconstruction movies of 293T-STING-GFP cells labeled with ER-Tracker Red.
